# Corrosion Behavior of Nitrogen-Containing Low-Nickel Weld Cladding in KCl-MgCl_2_ Eutectic Molten Salt at 900 °C

**DOI:** 10.3390/ma15248831

**Published:** 2022-12-10

**Authors:** Taisen Yang, Yunhai Su, Zhiyong Dai, Yingdi Wang, Xuewei Liang, Zuyong Wei

**Affiliations:** School of Materials Science and Engineering, Shenyang University of Technology, Shenyang 110870, China

**Keywords:** nitrogen-containing low-nickel weld cladding, high-temperature corrosion, chloride molten salt

## Abstract

In this paper, the element nitrogen (N) is used to partially replace the element nickel (Ni) in flux-cored wire. A 44%Ni-24%Cr-0.18N nitrogen-containing low-nickel flux-cored wire with excellent corrosion resistance is prepared. The corrosion behavior of nitrogen-containing low-nickel weld cladding and Inconel 625 weld cladding in 40 KCl + 60 MgCl_2_ (wt%) molten salt at 900 °C is studied. The results show that the selective dissolution of Cr occurs in both weld claddings. The corrosion resistance of the 44%Ni-24%Cr-0.18N nitrogen-containing low-nickel weld cladding is better than that of the Inconel 625 weld cladding. The reason is that added N can react with H^+^ in molten salt to generate ${\mathrm{NH}}_{4}^{+}$, remove corrosive impurities of MgOH^+^ in molten salt and change the corrosion environment. N preferentially combines with Cr to form Cr_2_N, reduces the diffusion precipitation of Cr and improves the corrosion resistance.

## 1. Introduction

Due to environmental pollution and the limitations of fossil fuel resources, solar energy has become an important part of human energy utilization [1]. Modern solar energy technology can be affected by night, dust and clouds and cannot work continuously, which greatly reduces the efficiency of solar devices [2]. In order to solve such problems, it is necessary to configure a set of energy storage devices to ensure continuous energy output. At present, nitrate is the main high-temperature energy storage medium. 

The use target of the next generation of high-temperature energy storage media in solar-power-generation applications is 600–900 °C, which exceeds the stable temperature of nitrate [3]. At high operating temperatures of solar power plants, 40 KCl + 60 MgCl_2_ (wt%) molten salt has the advantages of low price, high density, high boiling point, low saturated steam pressure and good thermal stability. It is suitable for solar thermal power storage materials for use in tower and parabolic trough systems. However, chloride molten salt is highly corrosive; therefore, the corrosivity of Cl^−^ must be considered when designing energy storage materials [4]. 

Chloride molten salt as an energy storage medium needs to work at high temperature, and thus its bearing equipment is mainly made of high-temperature-resistant materials. According to Sun et al. [5], Inconel 625 alloy has good high-temperature corrosion resistance, and it is widely used in solar heat storage tubes. In order to explore the corrosion mechanism between Inconel 625 alloy and chloride energy storage medium, a large number of researchers have conducted relevant research. 

Li, J.Y. [6] studied the corrosion behavior of 316 stainless steel and Inconel 625 alloy in ZnCl_2_, KCl and NaCl at 900 °C. He found that the two alloys were severely corroded in ternary chloride. A loose oxide layer was formed on the surface of the two alloys, and the oxide film did not protect the alloys. Ma, H.F. [7] found that the corrosion rate of Inconel 625 alloy in alkaline earth metals was greater than that in alkali metals. Inconel 625 alloy needs to face the corrosion of molten salt during service, resulting in corresponding corrosion failure. If the corrosion pit exceeds the standard, it needs to be repaired to ensure the continued service of the product. 

Inconel 625 flux-cored wire is the best choice for repairing castings. In recent years, the scarcity of nickel resources has led to the continuous rise in the price of Inconel 625 flux-cored wire. This has caused a large economic burden for solar power plants. In order to solve the problem of tight supply and high cost of nickel resources, it is urgent to find an element that can replace nickel. Through the literature, we found that N is a highly stable and austenitizing element [8]. At the same time, it can effectively expand the range of the austenite zone in steel. Based on the design concept of low-nickel flux-cored wire and high-nitrogen steel, N is suggested to partially replace Ni in flux-cored wire.

In this paper, low-cost N is used to replace part of the Ni content. On the basis of the composition of Inconel 625 flux-cored wire, we reduce the content of Ni by 20%, increase the content of Cr, Fe, Mn, Al and W elements to balance and adjust the alloy powder and design a nitrogen-containing low-nickel flux-cored wire. Taking the corrosion performance of weld cladding prepared by Inconel 625 flux-cored wire as a reference, the corrosion environment in a solar energy heat storage pipe is simulated. 

The high-temperature molten-salt corrosion depth and corrosion morphology of nitrogen-containing low-nickel weld cladding and Inconel 625 weld cladding in 40 KCl + 60 MgCl_2_ (wt%) at 900 °C are tested using the static immersion corrosion method. We explore the role of N in corrosion, provide theoretical guidance for the preparation of new welding materials with high-temperature corrosion performance not lower than that of nickel-based flux-cored wire and provide a material reference for the repair of solar energy heat storage pipes.

## 2. Materials and Methods

### 2.1. Experimental Materials and Methods

Schaeffler deeply studied the influence of alloy elements on the structure of stainless steel, converted it into the corresponding nickel equivalent Ni_eq_ and formed the famous Schaeffler diagram. However, this does not consider the effect of nitrogen. Speedel, M.O. [9] studied the effects of N on Ni equivalent (Ni_eq_) and corrected Ni_eq_, providing guidance for the composition design of high-nitrogen stainless steel. 

According to the modified nickel equivalent (Ni_eq_) calculation Formula 1, every increase of 1 wt% N is equivalent to an increase of 18 wt% Ni [8]. Co and C can also replace Ni to stabilize austenite. The increasing use of Co in batteries will also lead to higher prices in the next decade, and Co can cause allergic reactions, while C can enhance the formation trend of carbide and reduce corrosion resistance. It can be seen from Formula 1 that increasing the content of Mn cannot replace Ni but can reduce the Ni equivalent; therefore, N is the most reasonable element to replace Ni.

(1) $${\mathrm{Ni}}_{\mathrm{eq}}=\mathrm{Ni}\%+\mathrm{Co}\%+0.1\mathrm{Mn}\%-0.24{\left(\mathrm{Mn}\%\right)}^{2}+18N\%+30C\%$$

This paper involves four groups of flux-cored wires. The first three groups are nitrogen-containing low-nickel flux-cored wires, and the three weld claddings were marked as 1, 2 and 3, respectively. The fourth group was the comparison welding wire (weld cladding of Inconel 625) and was recorded as 4. Referring to the composition of Inconel 625 flux-cored wire, the appropriate content of Ni was controlled at 44.0%, the range of Cr was 20.0–24.0%, and the range of N was 0.15–0.21%. Carbon sulfur analysis and chemical methods were used to test the elemental content of the welding wire. The main components of the four sets of flux-cored wires are shown in Table 1. The experimental material was the newly developed nitrogen-containing low-nickel flux-cored wire. 

By adding manganese nitride and chromium nitride alloy powder to the core powder, N was used to partially replace Ni. Ni, Cr, Fe, Mn, Mo, Co, Cu, Ti, Al, Nb and W powders with purities above 99.9% were selected to adjust their element ratio. The powder mixer was used to uniformly mix the powder. Inconel 718 strip was selected for the preparation of flux-cored wire. The diameter of the newly developed nitrogen-containing low-nickel flux-cored wire was 2.55 mm.

The method of MIG welding was used for surfacing on a Q235 substrate. The size of the Q235 substrate was 180 × 100 × 20 mm. The normative reference of Q235 steel is shown in Table 2. In order to avoid the influence of dilution of weld cladding, three layers of weld cladding with a height of 12 mm were prepared. The welding process parameters were as follows: the welding current was 180 A, welding voltage was 28 V, welding speed was 8 m/h, and gas flow was 10 L/min. 

In order to reduce the splash overflow of N during welding, the shielding gas used in this test was 97%Ar + 3%N_2_. According to Cheng J H [10], when 3%N_2_ was added, there were no pores in the weld cladding, and the N fixation effect was best. In the surfacing process, each layer of surfacing was equivalent to the heat treatment of the previous layer, which can play a role in refining the grain. Therefore, the particle size of the third layer was relatively large. 

At the same time, in order to avoid the influence of dilution, it was not acceptable to sample from the surface of the first layer; thus, the sample was taken from the second layer of the surfacing layer. The sampling size was 10 × 10 × 2 mm. The weld cladding samples were polished with 200#–2000# metallographic abrasive paper, cleaned with acetone ultrasonic and dried for standby.

We referred to the national standards for corrosion test ASTM G31-2012a “Standard Guide for laboratory immersion corrosion testing of metals”. The static immersion corrosion method was used to simulate the working conditions of a solar heat storage pipe at 900 °C, and this was compared with Inconel 625 weld cladding. The molten salt used in this experiment was 40 KCl + 60 MgCl_2_ (wt%) (with purity greater than 99.5%). 

**Figure 1:**
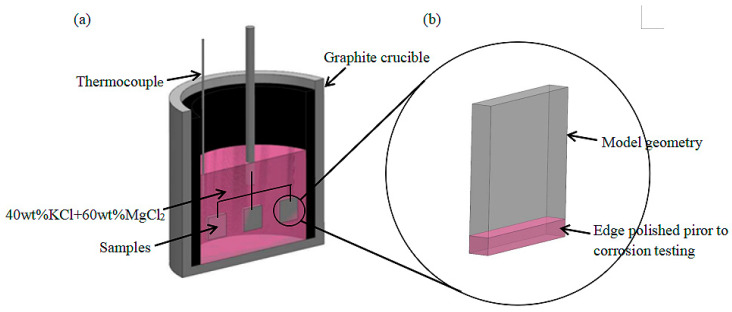
Schematic diagram of molten-salt corrosion. (**a**) Schematic diagram of the molten-salt corrosion test device and alloy sample. (**b**) Polishing area.

The salt mixture was dried in oven at 300 °C for 48 h inside a glove box, which was filled with high purity Ar (the H_2_O and O_2_ contents were maintained below 3 and 5 mg/kg, respectively), and was stored in the glove box before the corrosion test. For the high-temperature immersion corrosion test under an atmospheric environment, the thermocouple was placed in the drying tank to control the test temperature as shown in Figure 1a. The weld cladding was placed in the molten salt for high-temperature corrosion while ensuring that the molten salt completely covered the sample. 

It should be noted that the edges of the sample were polished before the high-temperature molten salt immersion corrosion test as shown in Figure 1b. The corrosion temperature was set at 900 °C, and the holding times were 10, 20, 30, 40, 50 and 60 h. After the molten salt test, the corroded samples were ultrasonically cleaned with distilled water and acetone to remove the molten salt and loosely adhered corrosion products. Before and after the corrosion test, we used a 0.1 mg electronic balance to weigh the samples and calculate the weight loss. In order to ensure the representativeness of the test data, three samples were used in each group, and the test results are the average values.

### 2.2. Properties of Weld Cladding

Before high-temperature molten-salt corrosion, the weld cladding layer was cut into small pieces of 10 × 10 × 10 mm, and then we used 200#–2000# abrasive paper to polish the surface of the weld cladding. We then used water-soluble diamond grinding paste with a particle size of 1.5 to mechanically polish the sample. The treated samples were etched with 50 g CuCl_2_ + 100 mL alcohol + 100 mL concentrated hydrochloric acid for 25–35 s. After corrosion, the metallographic samples were immediately washed with tap water, then wiped with absolute ethanol and finally dried with a hair dryer for standby. 

The microstructure of weld cladding was observed using a S-3400N scanning electron microscope, and the accelerating voltage was 30 KV. A JEM-2100 transmission electron microscope (TEM) was used to characterize the precipitated phase structure of the weld cladding to determine the influence of precipitation on the relative properties. The specific preparation process of TEM samples was as follows: a thickness of 600 μm was cut from the weld cladding and finally manually ground to 50 μm. 

A puncher was used to prepare Φ 3 mm flakes, and then we ground off the burrs with 5000# sandpaper. A TJ100-SE electrolytic double jet thinning instrument was used to prepare the transmission samples. The thinning of the sheet outlet automatically stopped. The electrolyte was a mixture of alcohol and perchloric acid with a volume ratio of 9:1. The temperature was about −25 °C, the current was 120 mA, and the voltage was 39 V.

After the high-temperature molten salt immersion corrosion, the phase composition of the weld cladding was measured using an XRD-7000. The specific parameters were as follows: a pure Cu target, the tube voltage was 40 kV, the current was 30 mA, the scanning speed was 2°/min, and the scanning range was 20–90°. Cross-section specimens of welding cladding were prepared by using standard grinding and polishing procedures. A Geminisem 300 scanning electron microscope and EPMA-1720 electron probe were used to analyze the morphology and element distribution of the cross section of the weld cladding.

## 3. Experimental Results

**Figure 2:**
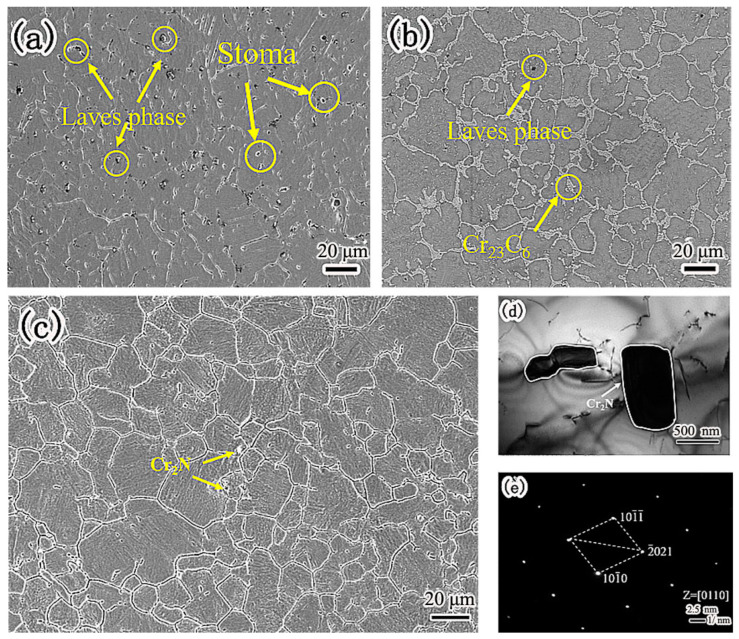
The microstructure of nitrogen-containing low-nickel weld cladding. (**a**) 1 nitrogen-containing low-nickel weld cladding precipitates; (**b**) 2 nitrogen-containing low-nickel weld cladding precipitates; (**c**) 3 nitrogen-containing low-nickel weld cladding precipitates; (**d**) Bright field image; (**e**) Diffraction spot.

Figure 2 is the microstructure of nitrogen-containing low-nickel weld cladding. There were many massive precipitates of nitrogen-containing low-nickel weld cladding 1, which were arranged irregularly as shown in Figure 2a. According to the energy spectrum analysis in Table 3, the irregular precipitates contained a great deal of Ni, Cr, Mo and Nb elements. After calculation, the sum of the Ni, Fe and Cr atomic percentages was 58.48%. The sum of the Nb, Mo and Ti atomic percentages was 29.61%. The precipitates approximately conform to A_2_B and have Laves (Ni, Fe, Cr)_2_(Nb, Mo, Ti) structure. Therefore, it is judged that this phase is Laves phase. 

In Figure 2a, the N content is 0.21%. It can be seen from Figure 2a that there are blowholes. In welding, the blowholes of the weld cladding have a great impact on the corrosion performance. Figure 2b shows the microstructure of nitrogen-containing low-nickel weld cladding 2. According to the energy spectrum analysis of the precipitates, the precipitates are Laves phase + Cr_23_C_6_. In Figure 2b, the content of N is 0.15%, the content of N is small, and no supersaturated N reacts with Cr to generate Cr_2_N. N is dissolved in the matrix. 

In nitrogen-containing low-nickel weld cladding 3, the content of N was 0.18%, and the supersaturated N combined with Cr to form Cr_2_N. There is discontinuous precipitation along the grain boundary as shown in Figure 2c. Through energy spectrum analysis, the atomic ratio of Cr to N was found to be 2:1, which shows that the flake precipitate is Cr_2_N. In order to further explore the material of the precipitated phase, the crystal structure was analyzed by TEM. Figure 2d shows the TEM morphology of grain boundary precipitates in nitrogen-containing low-nickel weld cladding 3. It can be seen from Figure 2e that the precipitates had a hexagonal structure. After calibration, the spot of massive precipitate was Cr_2_N, and the crystal band axis was [0110]. 

**Figure 3:**
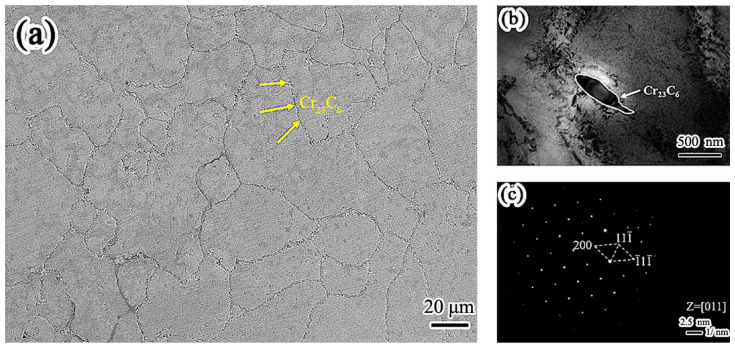
Microstructure of the Inconel 625 weld cladding. (**a**) Precipitates of the Inconel 625 weld cladding. (**b**) Bright field image. (**c**) Diffraction spot.

Figure 3 is the microstructure of the Inconel 625 weld cladding. In contrast, continuous precipitation was observed at the grain boundary of the Inconel 625 weld cladding as shown in Figure 3a. Figure 3b shows the TEM morphology of grain boundary precipitates in Inconel 625 weld cladding. The morphology of the precipitates is strip. It can be seen from Figure 3c that the precipitates along the grain boundary have face-centered cubic (FCC) structure. After calibration, the spot was Cr_23_C_6_, and the crystal band axis was [011]. 

By comparing the microstructure of the weld claddings, it can be seen that both of them are austenite phase; however, the precipitates are different. This is the reason that the corrosion resistance of the weld claddings is different. Cr_2_N precipitated from nitrogen-containing low-nickel weld cladding will play a positive role in resisting high-temperature molten-salt corrosion.

**Figure 4:**
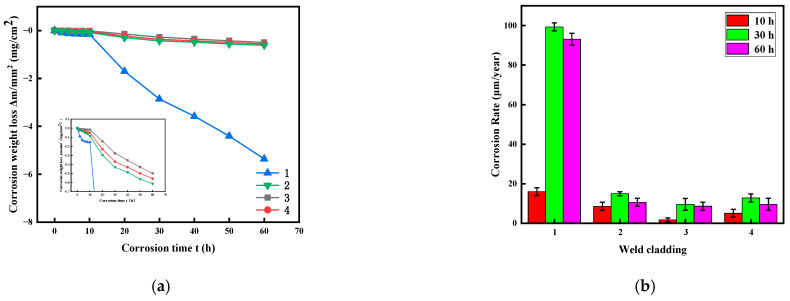
Molten-salt corrosion curve of nitrogen-containing low-nickel weld claddings and Inconel 625 weld cladding. (**a**) Corrosion weight loss curve. (**b**) Corrosion rate diagram.

Figure 4 is the corrosion weight loss curve and corrosion rate diagram of weld claddings 1, 2, 3 and 4. It can be seen from Figure 4a that the corrosion weight loss of weld claddings 1, 2, 3 and 4 shows an increasing trend with the increase in corrosion time. However, the increasing range is different for different time intervals. The corrosion weight loss of weld cladding 1 increased sharply from 0 to 60 h, and the corrosion amount was large. It can be seen from the local enlarged drawing that the corrosion amount of weld claddings 2, 3 and 4 increased slowly before 10 h, and the corrosion weight loss increased sharply after 10 h. 

We further clarify the influence of corrosion products on the corrosion rate. Before and after corrosion, the samples were weighed with an electronic balance (with an accuracy of 0.1 mg). Under the condition of uniform corrosion, the corrosion rate was calculated according to Formula (2).Weld claddings with corrosion times of 10, 30 and 60 h were selected to calculate the corrosion rate. The results are shown in Figure 4b. It can be seen from the corrosion rate diagram that the corrosion rates of weld claddings 1, 2, 3 and 4 after 60 h of corrosion were 93.15, 10.63, 8.65 and 9.65 μm/year. 

The corrosion rates of other weld claddings were low except for weld cladding 1. This is because the precipitated phase of weld cladding 1 was mainly Laves phase, and there were air holes; thus, the high-temperature corrosion resistance was poor. The maximum acceptable corrosion rate combined with solar-power-generation application is generally 30 µm/year, and the weld claddings 2, 3 and 4 meet the requirements of engineering applications. The corrosion rate of weld cladding 3 was lower than that of weld cladding 4, which indicates that it is feasible to increase N and reduce Ni in flux-cored wire.

(2) $${\mathrm{CR}}_{\mathrm{corr}}=\frac{365\times 10^{4}\times (m-m_{t})}{S_{1}\times t\times \rho }$$

CR_corr_ is the uniform corrosion rate (μm/year), m is the mass of the sample before immersion corrosion (g), m_t_ is the mass of the sample after immersion corrosion (g), S_1_ is the total area of the immersed sample (cm^2^), t is the immersion corrosion test time (h), and ρ is the density of the metal (g/cm^3^).

In the corrosion process, with an increase in the corrosion time, the contact time between the weld-cladding surface and molten salt is prolonged, the corrosion reaction is intensified, and the products are increased. Most corrosion products are brittle oxides or compounds. In the subsequent cleaning process, the matrix cracks and peels off, resulting in an increase in the corrosion rate after 10 h. We found that 10 h was the abrupt change point of the corrosion behavior of weld claddings through the above curve of corrosion weight loss with time. 

**Figure 5:**
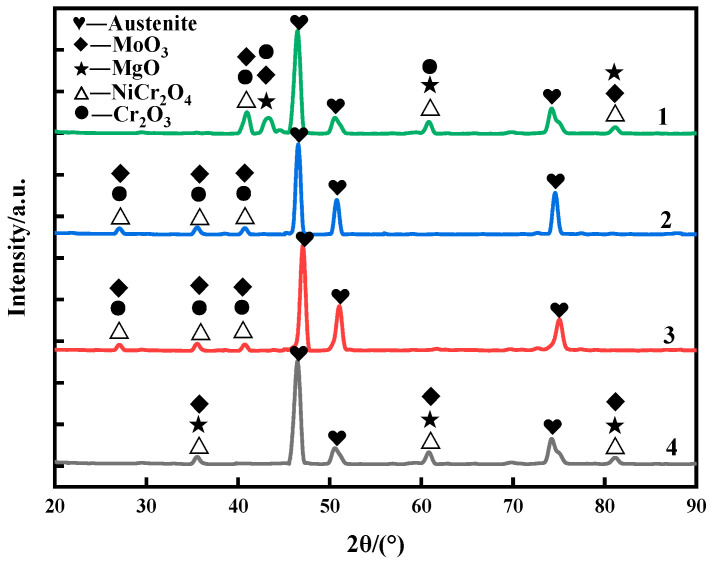
Surface phase composition of the nitrogen-containing low-nickel weld claddings and Inconel 625 weld cladding after corrosion for 10 h (1. 2. 3 are nitrogen-containing low-nickel weld claddings, and 4 is Inconel 625 weld cladding).

Therefore, the state of weld cladding after 10 h of corrosion was selected to analyze the corrosion products and corrosion mechanism. Figure 5 shows the surface phase composition of the four weld claddings after 10 h corrosion. It can be seen that the corrosion reaction was intense at 900 °C, and the weld claddings all correspond to the austenitic matrix. The corrosion products of nitrogen-containing low-nickel weld claddings 1, 2 and 3 contained MoO_3_, NiCr_2_O_4_ and Cr_2_O_3_. The synergistic effect of N and Mo can stabilize the Cr_2_O_3_ protective layer [10]. 

There is MgO precipitate on the surface of nitrogen-containing low-nickel weld cladding 1. MgO is unstable at high temperatures and will dissolve and fall off, which is the reason for the poor corrosion resistance of nitrogen-containing low-nickel weld cladding 1. Weld cladding 4 was only NiCr_2_O_4_, MoO_3_ and MgO. After corrosion, no Cr_2_O_3_ was detected on the surface of the Inconel 625 weld cladding, which means that the Cr_2_O_3_ protective film dissolved and fell off into the molten salt. 

It can be seen from the local enlarged figure of corrosion loss and XRD that the corrosion resistance of nitrogen-containing low-nickel weld cladding 3 was better than that of the Inconel 625 weld cladding; therefore, 3 is the best formula for designing nitrogen-containing low-nickel flux-cored wire. Therefore, the corrosion mechanism of nitrogen-containing low-nickel weld cladding 3 and the Inconel 625 weld cladding will be discussed in the follow-up.

**Figure 6:**
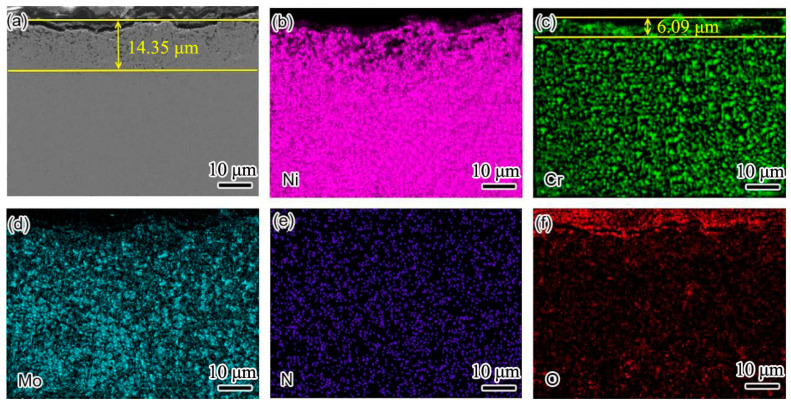
The cross-sectional element distribution of 3 nitrogen-containing low-nickel weld claddings after corrosion for 10 h. (**a**) Cross section morphology of 3 nitrogen-containing low-nickel weld claddings; (**b**) Ni element distribution; (**c**) Cr element distribution; (**d**) Mo element distribution; (**e**) N element distribution; (**f**) O element distribution.

**Figure 7:**
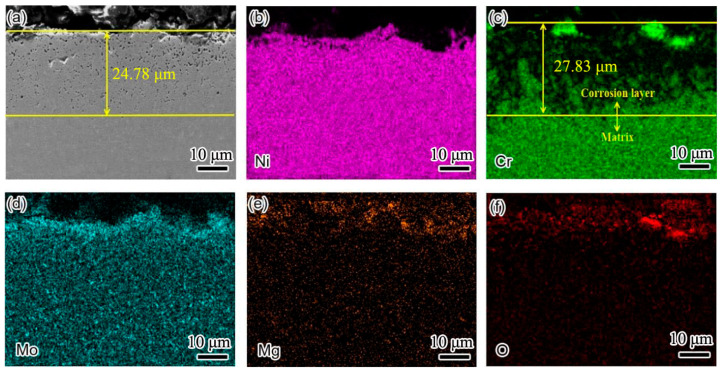
The cross-sectional element distribution of the Inconel 625 weld cladding after corrosion for 10 h. (**a**) Cross section morphology of Inconel 625 weld cladding; (**b**) Ni element distribution; (**c**) Cr element distribution; (**d**) Mo element distribution; (**e**) Mg element distribution; (**f**) O element distribution.

In order to clearly show the element distribution law of the two weld claddings sections, the cross sections of the two weld claddings after corrosion for 10 h were scanned and analyzed. The results are shown in Figure 6 and Figure 7. Figure 6 shows the cross-sectional element distribution of nitrogen-containing low-nickel weld cladding 3 after corrosion for 10 h. The distribution of Ni, Cr, N and Mo was uniform at 900 °C. The Cr diffused to the surface of the weld cladding and formed a 6.09 μm thick diffusion layer. 

According to XRD analysis and the element distribution in Figure 6, we determined that a Cr_2_O_3_ protective layer was formed on the weld-cladding surface. In addition, combined with the XRD results, we also found NiCr_2_O_4_ with a spinel structure on the weld-cladding surface. In the progress of corrosion reaction, the slight loss of Mo in the corrosion layer also shows that Mo elements migrated from the inside to the outside of the weld cladding. Mo elements first form a layer of MoO_2_ at the interface of the weld cladding, and the MoO_2_ oxide layer is oxidized to MoO_3_ after cracking [11]. 

D’Souza B [12] proposed that, during the corrosion process, Mo can promote the corrosion inner layer to produce a large amount of oxygen, forming a stable Cr_2_O_3_ protective layer, which is consistent with the above results. According to Figure 6e, N is dispersed in the weld cladding, and N is also enriched outside the corrosion layer. According to Wu, H.L. et al. [9], N is an active element in the interface. It is easy to aggregate at the grain boundary, and some elements in the weld cladding can accelerate the dissolution of N, such as Cr, Mn and Mo. 

Therefore, N is easy to combine with Cr and can precipitate at the grain boundary at an appropriate temperature to form Cr_2_N. It is clear that N participates in the corrosion resistance reaction of the weld-cladding surface and greatly improves the corrosion resistance of nitrogen-containing low-nickel weld cladding. This result is consistent with the results obtained in [11].

Figure 7 shows the cross-sectional element distribution of the Inconel 625 weld cladding after corrosion for 10 h. By comparing the corrosion depth in Figure 6, the corrosion depth increased from 14.35 to 24.78 μm. This shows that the corrosion resistance of the weld cladding was improved by adding N. At 900 °C, the Inconel 625 weld cladding corroded for 10 h. The Inconel 625 weld cladding produced a 27.83 μm Cr-poor layer, which was also the main reason for the decline of its corrosion resistance. At 900 °C, there was still a small amount of Mg on the surface of the Inconel 625 weld cladding. According to XRD analysis, the surface corrosion products were mainly spinel NiCr_2_O_4_, MoO_3_ and a small amount of MgO precipitation.

## 4. Discussion

**Figure 8:**
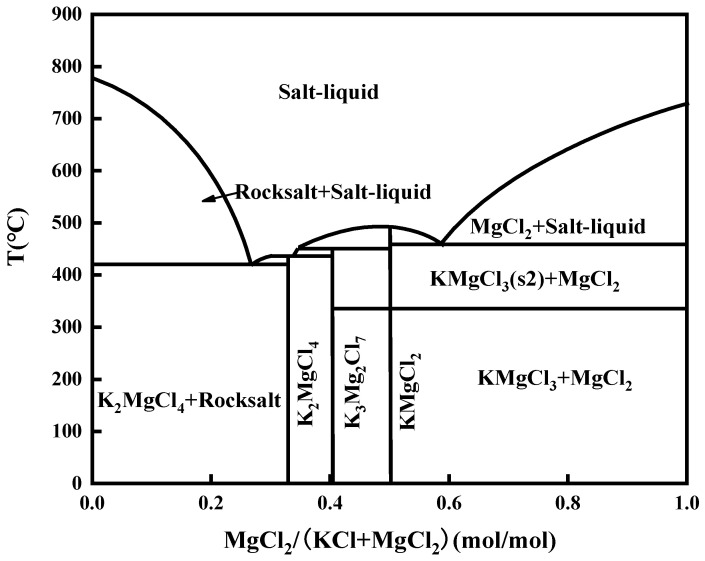
KCl + MgCl_2_ phase diagram.

The transition from solid molten salt to liquid molten salt is defined as a sharp increase in metal mass loss. It can be seen from Figure 4 that the abrupt corrosion point of weld claddings 3 and 4 was 10 h. This can be explained by two different states of molten salt. According to the KCl + MgCl_2_ phase diagram, the mixed molten salt is liquid at the test temperature as shown in Figure 8.

In order to understand the mechanism of chloride molten-salt corrosion of nitrogen-containing low-nickel weld cladding and Inconel 625 weld cladding at high temperature, the influence of N on corrosion, influence of Cr diffusion on corrosion and corrosion sensitivity are analyzed and expounded.

### 4.1. Effects of N on Corrosion

According to the acid-consumption model, during the corrosion process, the impurities in the chloride molten salt have a significant impact on the corrosion of the weld cladding, and the impurities mainly come from the water and oxygen in the molten salt. Due to the strong hydrophilicity of MgCl_2_ molten salt, hydrolysis can occur through Formula (3), and corrosive impurities of MgOH^+^, H^+^ and Cl^−^ can be produced. 

MgOH^+^ will further decompose to generate Mg^2+^, O^2-^ and H^+^, as shown in Formula (4) and will further corrode the weld cladding [13]. The N in the nitrogen-containing low-nickel weld cladding will undergo reaction (5) [14,15]. In this case, the presence of N will consume the H^+^ concentration, thus, increasing the PH value, indirectly removing MgOH^+^, improving the local corrosion environment and enhancing its corrosion resistance.

(3) $${\mathrm{MgCl}}_{2}\cdot H_{2}O\to {\mathrm{MgOH}}^{+}+H^{+}+2{\mathrm{Cl}}^{-}$$

(4) $${\mathrm{MgOH}}^{+}={\mathrm{Mg}}^{2+}+O^{2-}+H^{+}$$

(5) $$\left[N\right]+4H^{+}\to {\mathrm{NH}}_{4}^{+}$$

When designing the nitrogen-containing low-nickel weld cladding, N is mainly added through chromium nitride alloy. The main components of chromium nitride alloy are Cr_2_N and CrN. The decomposition reaction equation of Cr_2_N and CrN as well as the Gibbs free energy of formation of the reaction are reactions (6) and (7):

(6) $$2{\mathrm{Cr}}_{2}{N=4\mathrm{Cr}+N}_{2}\cdot \Delta_{f}G_{m}^{\theta }=198,400-93.98T$$

(7) $$2{\mathrm{CrN}=2\mathrm{Cr}+N}_{2}\cdot \Delta_{f}G_{m}^{\theta }=226,800-146.4T$$

Formulas (6) and (7) are inversely deduced to obtain Formula (8):

(8) $$2{\mathrm{Cr}}_{2}N=2\mathrm{CrN}+2\mathrm{Cr}\cdot \Delta_{f}G_{m}^{\theta }=-28,400+52.42T$$

According to Formula (8), the morphology of nitriding products depends on the temperature conditions. Only CrN can exist stably below 541.78 K, and Cr_2_N will be transformed into CrN. When the temperature is above 541.78 K, Cr_2_N exists stably, and CrN is transformed into Cr_2_N. When the corrosion temperature is 900 °C, chromium nitride exists in the form of Cr_2_N, which is consistent with the above TEM analysis. At 900 °C, Cr_2_N continue to react with O_2_, promoting the formation of a Cr_2_O_3_ protective film as shown in Formula (9) [16,17]. 

On the other hand, when facing Cl^−^ corrosion, the existence of Mo gives the Cr_2_O_3_ protective film the ability to repair. Mo and N in the weld cladding have synergistic effects, which can further improve the corrosion resistance of the weld cladding. The surface array formed by N and Mo inhibits the dissolution of the Cr_2_O_3_ protective film. Mo with high mobility acts with N near the corrosion layer. In the synergistic effect, N not only reduces the influence of pH value but also stabilizes molybdate, and molybdate also helps to form ammonia. At the same time, molybdate can also prevent the entry of corrosive ions, such as Cl^−^ and H^+^, protect Cr_2_O_3_ on the surface of weld cladding and improve the corrosion resistance of nitrogen-containing low-nickel weld cladding [18].

(9) $$2{\mathrm{Cr}}_{2}N+3O_{2}\to 2{\mathrm{Cr}}_{2}O_{3\;}+N_{2}↑$$

**Figure 9:**
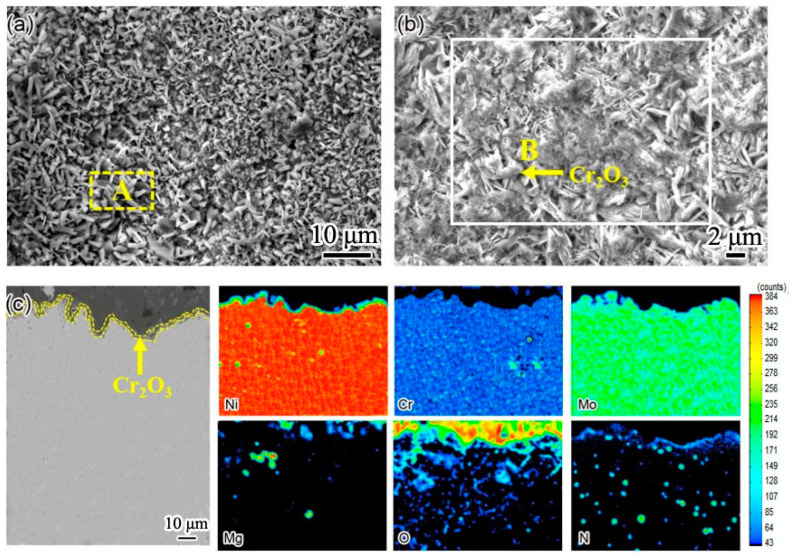
Surface and cross-sectional morphology of 3 nitrogen-containing low-nickel weld cladding after corrosion for 10 h. (**a**) SEM surface morphology. (**b**) High magnification of area A. (**c**) EPMA section morphology and corresponding elemental maps.

In order to further verify the positive effects of N on the Cr_2_O_3_ protective layer, we analyzed the surface and section of the corroded sample, and the results are shown in Figure 9. We enlarged area A in Figure 9a locally as shown in Figure 9b. The element content in the white area was tested using EPMA. After high-temperature molten salt immersion corrosion at 900 °C, the contents of Ni, Cr, Mo, Al and N on the surface of nitrogen-containing low-nickel weld cladding were 38.03%, 16.13%, 18.75%, 3.28% and 6.98%, respectively. The results are shown in Table 4. 

As shown in Figure 9b, layered Cr_2_O_3_ formed on the surface of nitrogen-containing low-nickel weld cladding. According to the element distribution of EPMA section in the Figure 9c, a Cr_2_O_3_ protective layer is formed on the surface of weld cladding. This indicates that the existence of N improves the stability of the Cr_2_O_3_ protective film and can inhibit the diffusion of Cr, which is consistent with the above analysis.

**Figure 10:**
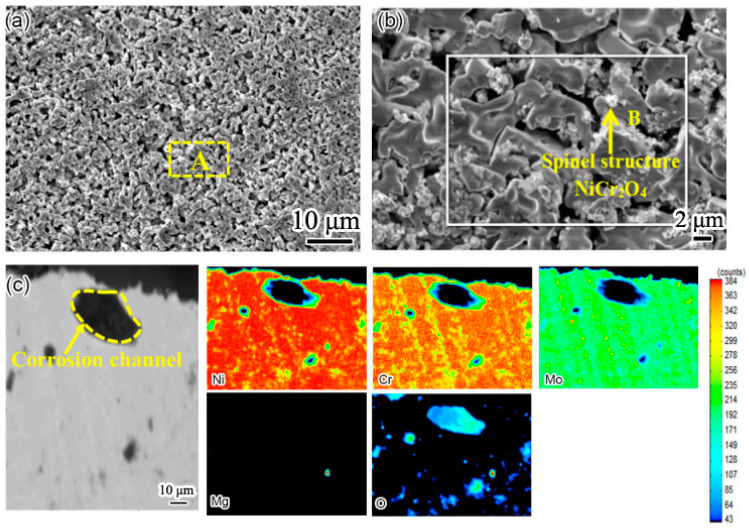
Surface and cross-sectional morphology of the Inconel 625 weld cladding after corrosion for 10 h. (**a**) SEM surface morphology. (**b**) High magnification of area A. (**c**) EPMA section morphology and corresponding elemental maps.

Compared with Inconel 625 weld cladding, Cr first combines with C to form Cr_23_C_6_ [19]. Inconel 625 weld cladding has a higher Cr content at the grain boundary, making it the best place for chloride molten-salt corrosion. Under a 900 °C corrosion environment, the grain boundary diffusion coefficient is greater than the lattice diffusion coefficient, which leads to the preferential dissolution of Cr into molten salt [20]. In order to further see the diffusion precipitation of Cr in Inconel 625 weld cladding, the surface and section of weld cladding were detected by EPMA, and the results are shown in Figure 10. 

According to Table 5, the contents of Ni, Cr, Mo and Mg on the surface of the Inconel 625 weld cladding were 46.24%, 15.91%, 11.33% and 0.04%, respectively. It can be seen from Figure 10b that the surface of the Inconel 625 weld cladding contained a large amount of NiCr_2_O_4_ with a spinel structure, which is the same as that of XRD. At 900 °C, the corrosion resistance of weld cladding was mainly reflected by the Cr and Mo elements. The contents of Cr and Mo of nitrogen-containing low-nickel weld cladding were higher than those of the Inconel 625 weld cladding, which also shows that the corrosion resistance of nitrogen-containing low-nickel weld cladding was better than the Inconel 625 weld cladding, which corresponds to the above corrosion weight-loss curve. 

It can be seen from Figure 10c that, after high-temperature corrosion, a large corrosion channel formed in the Inconel 625 weld cladding. According to the EMPA element distribution, there are a large number of oxides in the corrosion channel, which reduces its corrosion resistance [21]. In addition to the influence of oxides on corrosion resistance, the diffusion precipitation of Cr is also an important factor. 

The activity order of metal elements is Cr > Mo > Ni. The low content of Cr in Inconel 625 weld cladding is due to the poor stability of the Cr_2_O_3_ protective film in Inconel 625 weld cladding under a high-temperature corrosion environment. During the corrosion process, Cr_2_O_3_ generated by Cr oxidation is corroded and dissolved into molten salt; thus, the complete and continuous Cr_2_O_3_ protective film cannot be formed [22]. The surface content of Mo is rich in the two weld claddings; therefore, it is speculated that Mo has good stability in resisting molten-salt corrosion.

### 4.2. Effects of Cr Diffusion on Corrosion

According to the research results of Oak Ridge National Laboratory, the static immersion corrosion of nickel base alloy in molten salt is mainly affected by the diffusion rate of Cr [23]. The corrosion depth of weld cladding in the molten salt was much smaller than its thickness, and the content of Cr corroded in weld cladding was limited, while the quality of molten salt used in the corrosion experiment was large. Thus, the Cr concentration in molten salt after corrosion can be regarded as 0. It can be seen from [23] that the corrosion of weld cladding in molten salt can be determined by Fick’s second law. Combined with the Boltzman change and half-edge infinite-diffusion model, the distribution of Cr in the cross section of the Inconel 625 weld cladding matrix and corrosion layer follows Formula (10).

(10) $$c\left(x,t\right)=\frac{c_{0}}{2}\left[1-erf\left(\frac{x}{2\sqrt{Dt}}\right)\right]$$

**Figure 11:**
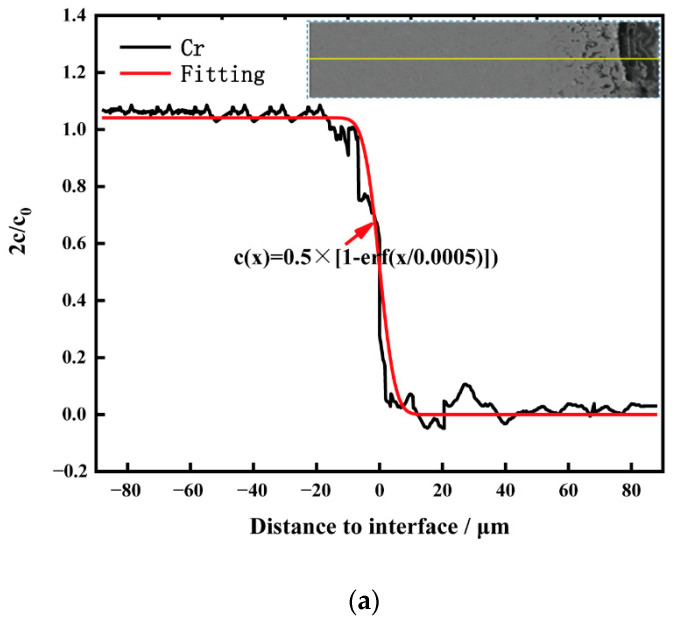

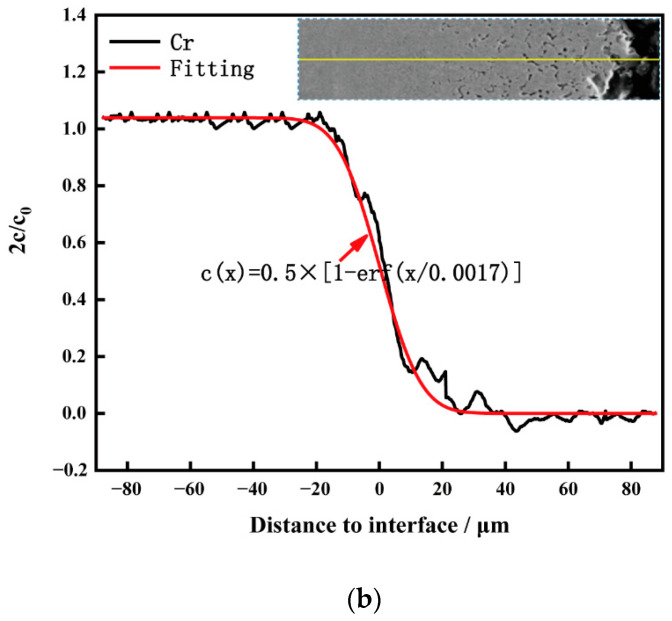
Element distribution of Cr between the matrix and corrosion layer in weld claddings. (**a**) Nitrogen-containing low-nickel weld cladding 3. (**b**) Inconel 625 weld cladding.

In Formula (10), *c* is the concentration of Cr in the weld cladding corrosion layer (g/cm^3^); *c*_0_ is the initial concentration of Cr in the weld cladding (g/cm^3^); *D* is the diffusion coefficient of Cr in the corrosion layer (cm^2^/s); *t* is the corrosion time (s); and *x* is the diffusion depth (cm). When *x* = 0, it is the interface between the weld cladding matrix and the corrosion layer. EPMA was used to conduct Cr line scanning on the cross section of cladding metal combined with Formula (10) after 40 KCl + 60 MgCl_2_ (wt%) molten-salt corrosion of nitrogen-containing low-nickel weld cladding for 10 h. The element distribution of Cr in the matrix and corrosion layer is shown in Figure 11a. 

Formula (11) can be obtained by formula fitting. According to the calculations, at 900 °C, after corrosion, the diffusion coefficient of Cr in nitrogen-containing low-nickel weld cladding is 2.9 × 10^−13^ cm^2^/s. Similarly, the diffusion coefficient of Cr in Inconel 625 weld cladding is 3.3 × 10^−12^ cm^2^/s as shown in Figure 11b and Formula (12). This is because Inconel 625 weld cladding generates Cr_23_C_6_ at the grain boundary, which increases the diffusion precipitation of Cr. Some studies showed that Cr is the main element in Cr_23_C_6_, and Cr_23_C_6_ preferentially forms at the grain boundary in the early stage, which is controlled by Cr diffusion. Once Cr_23_C_6_ nucleates, it needs Cr to support its growth. With the increase in time, Cr in grains gradually diffuses to the grain boundary to achieve dynamic balance. 

**Figure 12:**
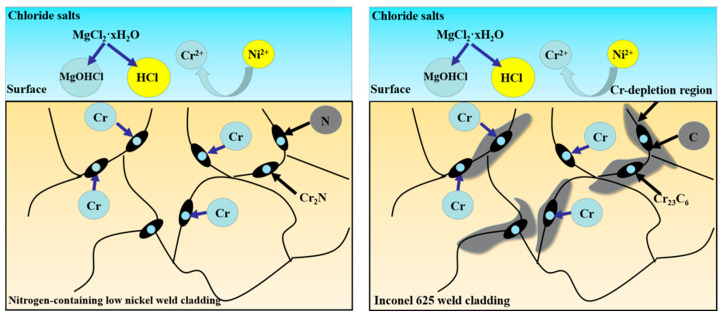
Action and corrosion mechanism of the element N.

This needs to consume a large amount of Cr to produce Cr-poor areas. In nitrogen-containing low-nickel weld cladding, Cr_2_N precipitates at the grain boundary, consuming less Cr, and there is no Cr-poor layer. According to the diffusion coefficient of Cr and Figure 9c, Cr formed a layer of dense Cr_2_O_3_ protective film on the surface of weld cladding. In addition to the positive effect of N, adding Mo, W and Al elements can strengthen the weld cladding by solid solution, thus, reducing the diffusion of Cr and reducing the corrosion of the weld cladding [24]. This is consistent with the above results of EPMA and the results of the Cr diffusion coefficient. The specific schematic diagram is shown in Figure 12.

(11) $$2\sqrt{Dt}=0.0005$$

(12) $$2\sqrt{Dt}=0.0017$$

### 4.3. Corrosion Sensitivity Analysis

The corrosion sensitivity factor (*CSI*) in Formula 13 is typically used to evaluate the sensitivity of the alloy to molten salt [25].

(13) $$CSI=\left(X_{Al}+X_{Cr}+X_{Ti}+X_{Nb}+X_{Hf}+X_{Ta}\right)/\left(X_{Ni}+X_{Fe}+X_{Co}+X_{Mn}+X_{Mo}+X_{W}+X_{Rc}+X_{Ru}\right)$$

In Formula (13), *X* represents the mole fraction of each element in the alloy. The lower the corrosion sensitivity factor, the better the molten-salt corrosion resistance. According to Formula 13, the CSI of nitrogen-containing low-nickel weld cladding was 0.4307. The CSI of the Inconel 625 weld cladding was 0.334. Interestingly, the corrosion sensitivity factor of nitrogen-containing low-nickel weld cladding was higher than that of the Inconel 625 weld cladding; however, it showed slight uniform corrosion. This is because there was Al in the nitrogen-containing low-nickel weld cladding. 

As shown in Formula (13), Al is considered as a beneficial element to resist molten-salt corrosion. Therefore, during the selection of structural materials for the molten salt environment, not only should the content of Cr in the alloy be considered as the determining element but also Al should be considered as an important element. Appropriate Al should be added in the design of nitrogen-containing low-nickel weld cladding. During high-temperature corrosion, O^2−^ can enter the inner layer of corrosion through pores, promoting the formation of composite oxides. Elements such as Al and Cr have high affinity with O. In KCl + MgCl_2_ molten salt, the Cr_2_O_3_ film can be damaged; however, an increase in Al will protect the Cr_2_O_3_ film to an extent [26].

## 5. Conclusions

In this paper, we mainly studied the corrosion behavior of nitrogen-containing low-nickel weld cladding and Inconel 625 weld cladding in 40 KCl + 60 MgCl_2_ (wt%) molten salt at 900 °C. Through systematic analysis and comparison of the hot corrosion behavior of the two weld claddings, the following conclusions are drawn:

(1) Through the comparison of high-temperature static immersion corrosion experiments, the optimal formula of nitrogen-containing low-nickel flux-cored wire was found to be 44%Ni-24%Cr-0.18N. The corrosion resistance of nitrogen-containing low-nickel weld cladding at 900 °C was better than that of the Inconel 625 weld cladding. The corrosion depth of nitrogen-containing low-nickel weld cladding was 14.35 μm after corrosion for 10 h. The corrosion depth of the Inconel 625 weld cladding was 24.78 μm after corrosion for 10 h.
(2) The surface of nitrogen-containing low-nickel weld cladding was mainly Cr_2_O_3_, MoO_3_ and NiCr_2_O_4_ after corrosion for 10 h at 900 °C. Adding N can improve the stability of a Cr_2_O_3_ protective film, inhibit the diffusion and precipitation of Cr and change the local corrosion environment. This is conducive to the formation of a Cr_2_O_3_ protective film and improves its corrosion resistance.
(3) At 900 °C, the corrosion sensitivity coefficient of nitrogen-containing low-nickel weld cladding was 0.4307 after corrosion for 10 h. The corrosion sensitivity coefficient of the Inconel 625 weld cladding was 0.334 after corrosion for 10 h. Al and N are beneficial elements to resisting molten-salt corrosion. The diffusion coefficient of Cr in nitrogen-containing low-nickel weld cladding was 2.9 × 10^−13^ cm^2^/s. The diffusion coefficient of Cr in Inconel 625 weld cladding was 3.3 × 10^−12^ cm^2^/s. The diffusion dissolution of Cr is the main reason for the decrease in corrosion resistance.

## Figures and Tables

**Table 1:** Chemical composition of flux-cored wire (wt%).

Specimen	Ni	Cr	Fe	Mn	Mo	Co	Cu	Ti	Nb	Al	W	N	C
1	44.0	20.0	14.40	3.8	5.1	0.7	0.2	1.4	5.6	1.8	2.78	0.21	0.01
2	44.0	22.0	12.85	3.4	6.4	0.7	0.2	1.5	5.4	1.4	1.95	0.15	0.05
3	44.0	24.0	12.62	2.2	6.3	0.7	0.2	1.1	5.3	1.1	2.28	0.18	0.02
4	64.7	22.0	0.9	0.23	8.4	0	0.02	0.4	3.15	0	0	0	0.2

**Table 2:** The normative reference of Q235 steel.

Chemical Composition (Mass, wt%)						Tensile Strength R_m_/MPa	Yield Strength R_p_/MPa	Elongation A/%
C	Mn	Si	S	P	Fe			
≤0.22	≤1.4	≤0.35	≤0.05	≤0.045	Bal	370–500	235	≥25

**Table 3:** The chemical composition of the precipitated phase of weld cladding (at %).

Phase	Ni	Cr	Mo	Nb	Fe	Ti	C	Al	N
Laves phase	41.59	13.91	12.16	14.14	2.98	3.31	6.05	3.28	2.58
Cr_23_C_6_	21.07	37.55	16.72	0.81	3.28	4.49	6.25	6.55	3.28
Cr_2_N	38.41	16.13	17.81	6.43	4.11	4.49	2.09	3.55	6.98

**Table 4:** Surface element content of nitrogen-containing low-nickel weld cladding 3 after corrosion for 10 h.

Element	Ni	Cr	Mo	Nb	Fe	Al	W	V	N
Content (%)	38.03	16.13	18.75	11.43	4.11	3.28	1.03	0.26	6.98

**Table 5:** Surface element content of the Inconel 625 weld cladding after corrosion for 10 h.

Element	Ni	Cr	Mo	Nb	Fe	Ti	Mg	C
Content (%)	46.24	15.91	11.33	19.14	0.98	0.31	0.04	6.05

## Data Availability

The authors confirm that all relevant data are included in the article.
